# A Colorimetric Ag^+^ Probe for Food Real-Time Visual Monitoring

**DOI:** 10.3390/nano12091389

**Published:** 2022-04-19

**Authors:** Jiahang Yu, Jun Qi, Zhen Li, Huixin Tian, Xinglian Xu

**Affiliations:** Jiangsu Collaborative Innovation Center of Meat Production and Processing, Quality and Safety Control, College of Food Science and Technology, Nanjing Agricultural University, Nanjing 210095, China; 2019208009@njau.edu.cn (J.Y.); junqi86@hotmail.com (J.Q.); 2018808149@njau.edu.cn (Z.L.); hxtian98@163.com (H.T.)

**Keywords:** colorimetric Ag^+^ probe, self-assembly, Ag NPs, amines, food detection

## Abstract

Monitoring food quality throughout the food supply chain is critical to ensuring global food safety and minimizing food losses. Here we find that simply by mixing an aqueous solution of sugar-stabilized Ag^+^ and amines in an open vessel leads to the generation of Ag NPs and an intelligent evaluation system based on a colorimetric Ag^+^ probe is developed for real-time visual monitoring of food freshness. The self-assembly reaction between methylamine (MA) generated during meat storage and the colorimetric Ag^+^ probe produces different color changes that indicate changes in the quality of the meat. The colorimetric Ag^+^ probe was integrated into food packaging systems for real-time monitoring of chilled broiler meat freshness. The proposed evaluation system provides a versatile approach for detecting biogenic amines and monitoring chilled broiler meat freshness and it has the advantages of high selectivity, real-time and on-site measurements, sensitivity, economy, and safety and holds great public health significance.

## 1. Introduction

Accompanied by the development of international trade and industrial production, food safety has gradually become an exacting global issue with public health implications due to the multiplicity and complexity of foods and food production systems [1]. Environmental factors such as a lack of cold storage facilities, improper handling, and improper transport can easily lead to food oxidation and spoilage microorganisms are one of the main reasons for food poisoning and food spoilage; this commonly produces off-odors, off-flavors, discoloration, slime [2] and especially toxic compounds such as biogenic amines (BAs) and ammonia [3]. Therefore, monitoring spoilage during food storage and transportation has been a matter of worldwide concern. Traditional freshness monitoring relies primarily on end-point inspections, which cannot judge freshness within the shelf life and cannot meet the demands of circulation and trade. A process for continuous monitoring of freshness has become imperative for the global food industry and intelligent packaging is the best choice for addressing this challenge. Early research developed specific techniques, including fluorescence spectrometry [4], Raman spectroscopy [5], and electrochemistry [6], to monitor the freshness of food. However, most methods are not suitable for real-time and on-site analysis and this strategy of indirect detection suffers from large deviations. Direct monitoring technology such as colorimetric sensors can reflect the process and intensity of food spoilage and more accurately monitor changes in freshness [7]. Amines (BAs and ammonia) directly reflect the freshness of food and real-time monitoring based on characteristic amines is an important breakthrough in monitoring freshness. The combination of amines and specific materials that present a color change distinguishable to the naked eye would represent a potential breakthrough for monitoring food freshness.

Colorimetric sensors are effective in achieving simultaneous identification of multiple analytes and predicting the components of unknown complex samples; they can perform simple, rapid, low-cost, real-time, and nondestructive detection of analytes with a portable system and without the need for complex instrumentation and thus have been widely used for safety and quality monitoring with a variety of industrial products, such as foods [8], drinks [9], environmental samples [10], or bioliquids [11]. Colorimetric sensors are designed to detect subtle changes by converting the relevant chemical or physical properties of molecular or ionic species into visible color changes observable by the naked eye. Much effort has therefore gone into the development of colorimetric sensors. Recently, significant progress has been made with nanostructure-based colorimetric sensors using a variety of interesting optical properties of nanostructures [12]. In particular, one of the most striking emerging trends is colorimetric sensing based on plasmonic coupling (PC) [13] and localized surface plasmon resonance (LSPR) [14] with silver nanoparticles (Ag NPs).

Self-assembly of nanoparticles offers great opportunities for bottom-up creation of functional materials and systems, which is a promising and practical pathway for creating stimulus-responsive nanomaterials with novel properties suitable for drug delivery [15], polymer synthesis [16] and sensing [17,18,19,20]. Wong et al. [16]. demonstrated a simple, virtually instantaneous route for making nanoparticles from curcumin in water simply by mixing fructose and curcumin, which showed great promise for self-assembly. One of these important applications involves the combination of modern sensing techniques and controlled self-assembly to regulate various self-assembly processes [21]. Ma et al. [18]. demonstrated a nanoplasmonic platform that relies on the lead-mediated assembly of glutathione-functionalized gold nanoparticles (NPs) for sensitive detection of lead ions. Wang and coworkers [19] presented self-assembly template-driven 3D inverse opal microspheres functionalized with catalyst nanoparticles that enable good acetone selectivity and high sensitivity at the ppb level. Zhang et al. [20]. demonstrated self-assembled microgel arrays for electrostatic concentration and surface-enhanced Raman spectroscopy used to detect charged pesticides in seawater. Although these self-assembled sensors have high selectivity and sensitivity, the acquisition of reflectance signals, resistive signals, or Raman spectra is demanding in terms of operating conditions, temperature, or external environment. There are many problems in applying these studies to food quality sensing, such as safety and convenience, which constitute limitations for practical application. To overcome these challenges, we have developed an efficient and convenient colorimetric silver ion (Ag^+^) probe. Amine-induced self-assembly of sugar-stabilized Ag NPs was used to produce colorimetric probes that produce color changes discernible to the naked eye, which can then be used for amine sensing. In the specific application, the colorimetric Ag^+^ probe was prepared simply by mixing D-ribose and AgNO_3_ in water. The self-assembly reaction between methylamine (MA) generated during meat storage and the colorimetric Ag^+^ probe produces Ag NPs of different colors that indicate changes in the quality of the meat. It is well known that traditional applications of sensors based on precious metal nanoparticles mainly involve the preparation of nanoparticles by physical methods (hydrothermal, solvent thermal, radiation, etc.) and chemical methods (sodium borohydride reduction, sodium hydroxide reduction, etc.) before further use for sensing and these methods suffer from complex preparation processes and poor safety, which limit their large-scale preparation and specific applications. In this study, we aimed at developing an amines sensitive colorimetric Ag^+^ probe, which is expected to provide the consumer with information about meat quality and safety without the need for user manuals or technical skills; the method is designed for consumers or low-skilled professionals because the probe responses are relatively easy to read. Our system operates in real-time and is accurate, economical, and nondestructive. For reasons of safety, the colorimetric Ag^+^ probe uses common biodegradable and biocompatible materials with good stability, is easily bio-functionalized, and operates under typical meat packaging conditions, it can be applied anywhere in the meat supply chain for real-time monitoring of meat freshness.

## 2. Materials and Methods

### 2.1. Materials

Silver nitrate (AgNO_3_), D-ribose, and trisodium citrate (TSC) were purchased from Shanghai Aladdin Bio-Technology Co., Ltd. (Shanghai, China). Methylamine solution (98%), NH_3_·H_2_O (25% in water), trimethylamine solution (45% in water), cadaverine (>98%), putrescine (>98%), tyramine (98%), sodium sulfide (Na_2_S), sodium hydrogensulfide hydrate (NaHS), methanol, N-pentanol, dithiothreitol (DTT), ethyl acetate (EA), acetic acid (AA), tetrahydrofuran (THF), sodium trichloroacetate (ST), sodium chloride (NaCl), N-heptanal, N-nonanal and 2-heptanone were purchased from Shanghai Yuanye Bio-Technology Co., Ltd. Tyvek paper and foam tape (1600T) were purchased from Beijing 3M Co., Ltd. (Beijing, China). Chilled broiler meat was purchased from Jiangsu Lihua Animal Husbandry Co., Ltd. (Changzhou, China).

### 2.2. Instruments

DLS and ζ-potential measurements were recorded on a Zetasizer Nano ZS90 (Zeta sizer Nano, Malvern, UK). UV–vis absorption spectra were measured on a SpectraMax M2 microplate reader (Molecular Devices, Sunnyvale, CA, USA). IR spectra (liquid infrared) were obtained by a Thermo Antaris II FTIR infrared spectrometer (Thermo Fischer Scientific, Waltham, MA, USA). Raman spectra were obtained using a Raman spectrometer (HORIBA, LabRAM HR Evolution, France) at 532 nm. Changes in color were recorded by a Huawei P30 mobile phone camera and TEM images were acquired using a JEM 2100F microscope (TEM, JEM-2100F, JEOL, Tokyo, Japan). Chilled broiler meat was packaged with a modified atmosphere packaging machine SMART500.

### 2.3. Synthesis of the Colorimetric Ag^+^ Probe

Sugar (0.5 g) was added to ultrapure water (84 mL) and stirred for 10 min. To this solution, AgNO3 (10^−2^ M, 10 mL) was added dropwise with gentle stirring (120  rpm), resulting in a final colorimetric Ag^+^ probe. The colorimetric Ag^+^ probes were stored in a refrigerator at 4 °C until use.

### 2.4. Preparation of Colorimetric Sensing Label

The colorimetric tank was cut from foam tape with a thickness of 2 mm. There was one round hole with diameters of 10 mm in the middle and the capacity was approximately 0.16 mL. The bottom was sealed with Tyvek paper, which has excellent breathability and safety.

The colorimetric Ag^+^ probe was added to the round holes and a 6 mm diameter black paper was pasted at the bottom right as the black background to obtain a colorimetric sensing label.

### 2.5. Detection of TVB-N

Total volatile basic nitrogen (TVB-N) was determined according to the China National Food Safety Standard methods—Method for analysis of hygienic standard of meat and meat products (GB5009.228-2016). TVB-N contents were expressed as mg of TVB-N per 100 g of chicken.

## 3. Results and Discussion

### 3.1. Preparation and Characterization of Colorimetric Ag^+^ Probe

**Figure 1:**
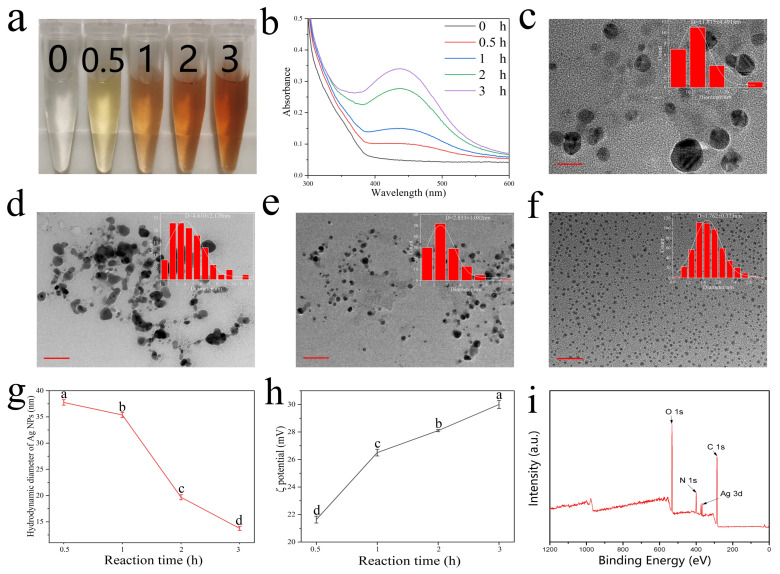
Digital photographs (**a**), UV–vis spectra (**b**) of Ag NPs at different reaction times; TEM image and particle size histogram of Ag NPs at different reaction times: (**c**) 0.5 h, (**d**) 1 h, (**e**) 2 h, and (**f**) 3 h (scale bar = 20 nm); hydrodynamic diameter (**g**) and zeta potential (**h**) of Ag NPs at different reaction times, vertical bars represent standard deviation of the mean (n = 6), significance was considered at *p* < 0.05; (**i**) XPS survey scan of Ag NPs reacted for 3 h.

Methylamine-induced self-assembly of Ag NPs used to produce a colorimetric Ag^+^ probe and the morphological changes of the Ag NPs at different reaction times were studied. The degree of self-assembly of Ag NPs varies with reaction time. This results in changes in the number of ligands bound per particle surface. Different quantities of ligands on the surfaces of NPs also determine the distances between particles, which, in turn, controls the plasmonic coupling of the Ag NPs and the color of the solution [22]. Equal amounts of colorimetric Ag^+^ probe and MA solution (80 µM) were quickly mixed under ambient conditions and the color of the reaction mixture changed progressively to deep yellow after different incubation times (0.5, 1, 2, and 3 h). The reaction time was set to 3 h (as optimized in Figure S1, Supporting Information). Without using any instrument, it was easy for the naked eye to distinguish the color change (Figure 1a). The UV–vis absorption peaks (400–480 nm) were characteristic of the Ag NPs surface plasmon resonance effect and consistent with the absorption peaks of Ag NPs in solution [23]. With increasing reaction time, new peaks appeared at ~440 nm and the peaks became sharper and more symmetrical (Figure 1b), confirming the formation of metal nanoparticles. The TEM images of Ag NPs were analyzed using the Nano Measurer 1.2 software to determine particles size distribution. The TEM image and particle size distribution histogram showed that with increasing reaction time, the particle sizes of the Ag NPs decreased and the distribution was more uniform (Figure 1c–f). The physicochemical properties of NPs are mainly explained by their hydrodynamic diameters and zeta potentials [24]. Dynamic light scattering (DLS) was used to measure the hydrodynamic diameters of the Ag NPs; the results suggested a drastic decrease in Ag NP size from 37.76 nm (1 h) to 13.75 nm (3 h) with increasing reaction time (Figure 1g). Prolonging the reaction time significantly promoted the growth and assembly of Ag NPs, which is consistent with the TEM observations. As shown in Figure 1h, the ξ potential of the Ag NPs changed from 21.63 to 30 mV when the reaction times ranged from 0.5 to 3 h, indicating an increase in surface charge and hence an increase in ligand coverage density on the Ag NP surface. This showed that when the Ag precursor and ligand contents were held constant in this reaction system, the Ag NPs progressively decreased in size and simultaneously increased the ligand density on their surfaces. The surface state of the Ag NPs was examined by X-ray photoelectron spectra (XPS), as shown in Figure 1i, further confirming the presence of C, N, O, and Ag in the Ag NPs. Based on these results, it can be concluded that extended reaction times significantly promoted the growth of Ag NPs.

**Figure 2:**
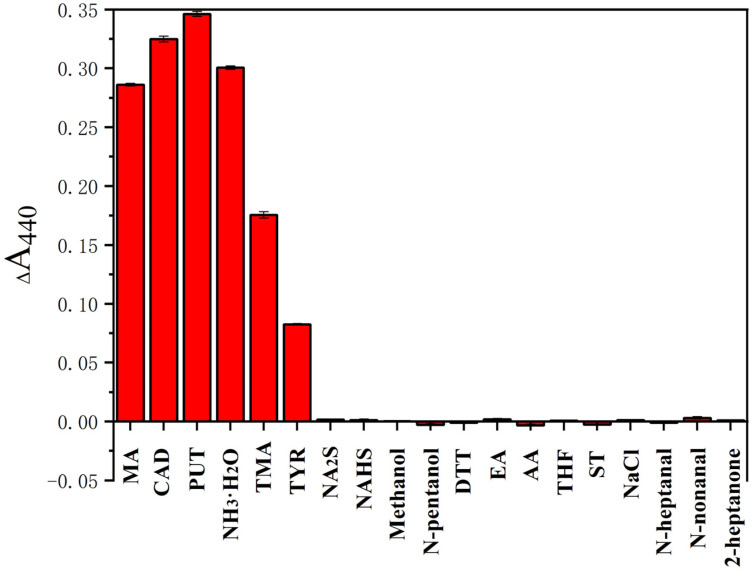
Changes in absorbance values at 440 nm measured before and after reactions between the D-Ag^+^ probe and various compounds (Methylamine (MA), cadaverine (CAD), putrescine (PUT), NH_3_·H_2_O, trimethylamine (TMA), tyramine (TYR): 8 × 10^−5^ M; sodium sulfide (Na_2_S), sodium hydrosulfide hydrate (Na HS), methanol, N-pentanol, dithiothreitol (DTT), ethyl acetate (EA), acetic acid (AA), tetrahydrofuran (THF), sodium trichloroacetate (ST), sodium chloride (NaCl), N-heptanal, N-nonanal and 2-heptanone, 1 × 10^−4^ M). Each data point is the mean of five replicate samples (mean ± standard deviation). Vertical bars represent the standard deviation of the mean. Significance was considered at *p* < 0.05.

To evaluate the selectivity of the colorimetric Ag^+^ probe, several potential interfering compounds were evaluated under the reaction conditions described above. As shown in Figure 2, the absorbance at 440 nm was measured before and after reactions between the colorimetric Ag^+^ probe and various compounds, which confirmed that amines produced significant absorbance changes at 440 nm. In contrast, no significant absorbance change was observed after the introduction of other compounds, and each compound was added at a concentration higher than that of the amines. It should be noted that the response of the colorimetric Ag^+^ probe to sodium sulfide, methanol, and n-heptanal was negligible. These compounds have a strong complexing ability that could have interfered with the colorimetric Ag^+^ probe. These results all demonstrate that the tested compounds have negligible effects on the colorimetric Ag^+^ probe and that the proposed method has high selectivity for amines.

### 3.2. Sensing Mechanism Exploration

**Figure 3:**
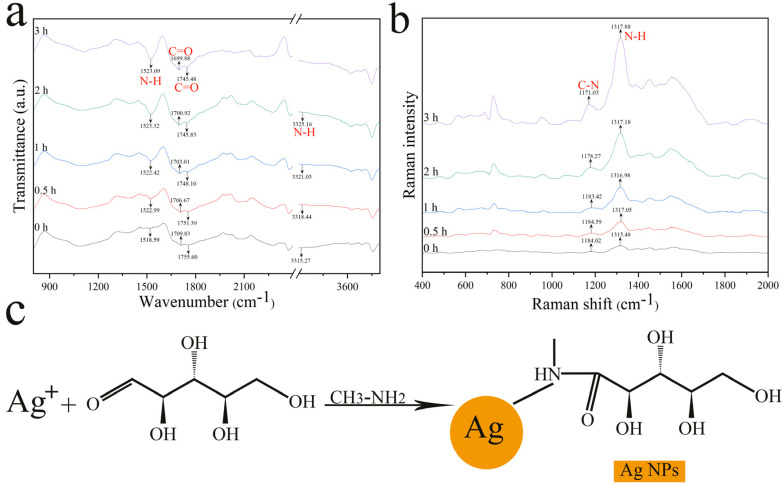
FTIR (**a**) and Raman (**b**) spectra for Ag NPs at different reaction times; (**c**) sensing mechanism of the proposed assay.

Changes in chemical bonding and functional groups before and after reaction were determined by Fourier transform infrared (FTIR) spectroscopy (Figure 3a). It is noteworthy that we characterized the same Ag NPs with different processing times to rule out interference from different batches. The main functional groups of D-ribose include hydroxyl and aldehyde groups. After mixing MA and the colorimetric Ag^+^ probe, upon oxidation of the aldehyde by Ag^+^, the –NH_2_ group was deprotonated to –NH and then formed an amide linkage with the aldehyde group of D-ribose [25,26]. The IR spectrum showed that the N–H stretches from primary amines appeared in the range 3300–3500 cm^−1^ as twin peaks [27]. The twin peaks for NH_2_ disappeared and only a single peak was observed at approximately 3300–3500 cm^−1^ as the reaction progressed, indicating that the primary amines were converted to secondary amines [28]. The peaks for primary and secondary amines in the range 3300–3500 cm^−1^ disappeared in the 3rd hour, which indicated a complete reaction of MA. The peak at 1755.6 cm^−1^ is characteristic of the aldehyde carbonyl of D-ribose and the band shifted upon the addition of MA; this indicated a chemical reaction between the aldehyde carbonyl and MA [29,30]. Additionally, new peaks attributed to amides appeared at 1522.99 cm^−1^ (amide II) and 1709 cm^−1^ (amide I). Furthermore, the formation of Ag NPs is accompanied by the shift, sharpening, and expansion of the IR spectra with the extension of reaction time. The separation between the amide I and amide II peaks also reflected interactions between N, O, and Ag atoms. These results are clear evidence of the involvement of amide groups in interactions with Ag NPs. The amide groups interact and coordinate with Ag^+^ ions adsorbed on the surface of Ag NPs, thereby precluding AgNP agglomeration and increasing stability.

Confocal Raman microscopy is a powerful tool for characterizing the surface functional groups capping Ag NPs (Figure 3b). The nature of Ag NPs gives rise to surface-enhanced Raman scattering (SERS) and the SERS signal is attributed to the enhancement of the electromagnetic field due to localized surface plasmon resonance (LSPR) and to interactions of the analyte with the SERS signal-enhancing surface [31]. Strong amide III (1171 cm^−1^) [32,33] and amide II (1317 cm^−1^) [34] Raman signals can be seen in Figure 3b, which may have originated from newly formed amide bonds. In particular, the signal intensity and sharpness of the characteristic peak at 1317 cm^−1^ were obviously enhanced with increasing reaction time (Figure 3), indicating that the NH moiety of the amide bond capped the Ag surfaces. The results are consistent with those obtained from FTIR spectra. The sensing mechanism of the proposed assay is shown in Figure 3c.

### 3.3. BAs-Response Behavior of Colorimetric Ag^+^ Probe

**Figure 4:**
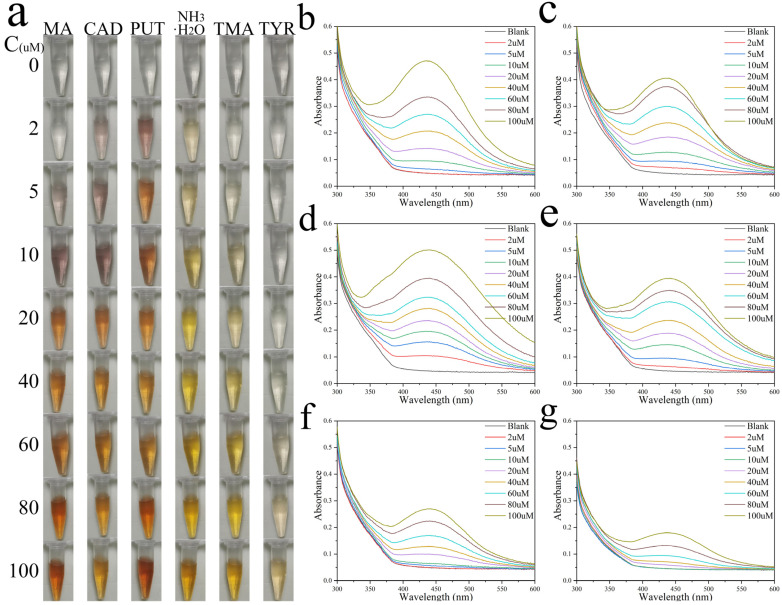
Discrimination among BAs with colorimetric Ag^+^ probe. (**a**) Digital photographs of a colorimetric Ag^+^ probe mixed with MA, CAD, PUT, NH_3_·H_2_O, TMA, and TYR solutions show different color combinations. The color of the colorimetric Ag^+^ probe changed as the solution concentration was increased from 2 to 100 µM. (**b**–**g**) UV–vis spectra of colorimetric Ag^+^ probe exposed to BAs solutions with different concentrations (**b**): MA, (**c**): CAD, (**d**): PUT, (**e**): NH3H2O, (**f**): TMA, (**g**): TYR.

To assess whether our colorimetric Ag^+^ probe could be used to monitor meat freshness, we tested the responses of the colorimetric Ag^+^ probe to multiple BAs typically detected in rotting meats, including MA, cadaverine (CAD), tyramine (TYR), trimethylamine (TMA), putrescine (PUT) and ammonia (NH3·H2O) [32,33,34], which are useful freshness indicators. Varying concentrations of amines were prepared in solutions and mixed with equal amounts of colorimetric Ag^+^ probe, the reaction time was set to 3 h. Due to the different structures of each BA, the colorimetric Ag^+^ probe triggered different self-assembly processes forming a specific color combination that represents each of the six BAs (Figure 4a). Figure 4b–d shows the UV–vis spectra of the colorimetric Ag^+^ probe exposed to BAs. The colorimetric Ag^+^ probe offers better visualization performance and sensitivity. More importantly, the colorimetric Ag^+^ probe showed a similar colorimetric response to other common BAs, thus providing the feasibility of monitoring food spoilage with total BAs content. This single-reaction colorimetric Ag^+^ probe array provided a simple and effective strategy for distinguishing compounds. Compared with previous techniques [35,36,37,38,39], the colorimetric Ag^+^ probe array exhibited multiple advantages: (i) it has a broader detection range; (ii) it has a high level of safety; (iii) it can identify a variety of amines; and (iv) it requires no complex synthetic procedures.

### 3.4. Immobilization of a Colorimetric Ag^+^ Probe in Colorimetric Tank as a Colorimetric Sensing Label

Although meats have complex chemical compositions, it is seldom necessary for real-world applications to gather information on hundreds of coexisting compounds. Moreover, accurate detection and differentiation of different components in highly complex mixtures is challenging with even sophisticated analytical techniques. The most crucial task is to analyze and identify changes in meat quality during processing, transportation, storage, and marketing. Therefore, highly differentiated colorimetric identification of the entire mixture is both quicker and more cost-effective. More importantly, the colorimetric Ag^+^ probes showed obvious colorimetric signal changes for different amines, thus indicating the feasibility of monitoring meat spoilage with total amine content. These colorimetric indicators need to verify meat quality with an accuracy comparable to existing quality indexes (e.g., TVB-N). To facilitate the broader application of the proposed method, the colorimetric Ag^+^ probe was combined with colorimetric tanks to produce a solid version of the colorimetric sensing label (Figure S2) and applied to chilled broiler meat in modified atmosphere packaging (20% CO_2_, 80% N_2_) to monitor changes in chilled broiler meat freshness.

**Figure 5:**
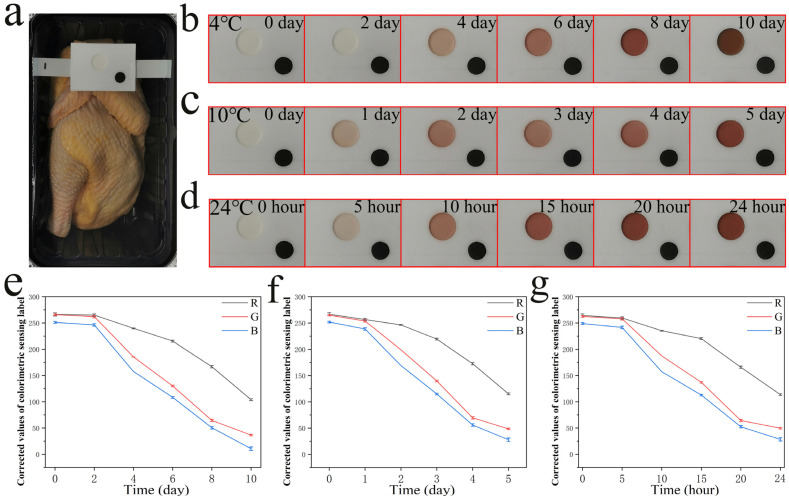
(**a**) The schematic of the modified atmosphere packaging (MAP: 20% CO_2_, 80% N_2_), (**b**–**d**) color change in colorimetric sensing labels in chilled broiler meat wrapped in modified atmosphere packaging at different temperatures (**b**): 4 °C, (**c**): 10 °C, (**d**): 24 °C; (**e**) *R*, *G*, and *B* values of colorimetric sensing label at 4 °C; (**f**) *R*, *G*, and *B* values of colorimetric sensing label at 10 °C; (**g**) *R*, *G*, and *B* values of colorimetric sensing label at 24 °C.

The colorimetric tank is made of foam tape and Tyvek paper keeps the colorimetric Ag^+^ probes from leaking or contacting the meats while still in contact with the amines produced in chilled broiler meat in modified atmosphere packaging. During the experiment on chilled broiler meat spoilage, the colorimetric sensing label was attached to packaging film inside the modified atmosphere packaging box (Figure 5a) and it showed a clear color change that was suitable for monitoring the freshness of chilled broiler meat with the naked eye. The packages were stored at different temperatures and mobile phone images were taken at fixed times without opening the packaging. The distinct color changes were suitable for monitoring the freshness of chilled broiler meat with the naked eye. As shown in Figure 5b–d, the colorimetric sensing label exhibited color changes from colorless to light red and dark red, similar to those observed in solution. The color change response was enhanced with increasing temperature, indicating that the colorimetric sensing label is suitable for monitoring chilled broiler meat quality at different temperatures. As there is normally a difference in ambient light conditions, imaging angle/distance, and cellphone brand when taking a photographic sample with a cell phone, a new method using white and black backgrounds is proposed for a colorimetric sensing label that can calibrate the colors obtained from the cell phone camera, thereby yielding accurate measurements [40]. The black and white backgrounds were, respectively, defined as the black paper pasted on the foam tape and white foam tape, the RGB values of the black paper at the center of the colorimetric sensing label were taken as a black background and the RGB values of foam tape next to the black paper were taken as a white background, as shown in Figure S2c. The digital RGB values of the colored spots on the colorimetric sensing label were corrected by Equation (1):

(1) $$R_{corr}=\left(\frac{256}{R_{w}-R_{b}}\right)\left(R_{meas}-R_{b}\right)$$

where *R_corr_* is the corrected value, *R_w_* is the white background value, *R_b_* is the black background value and *R_meas_* is the measured value of the colorimetric sensing label.

**Figure 6:**
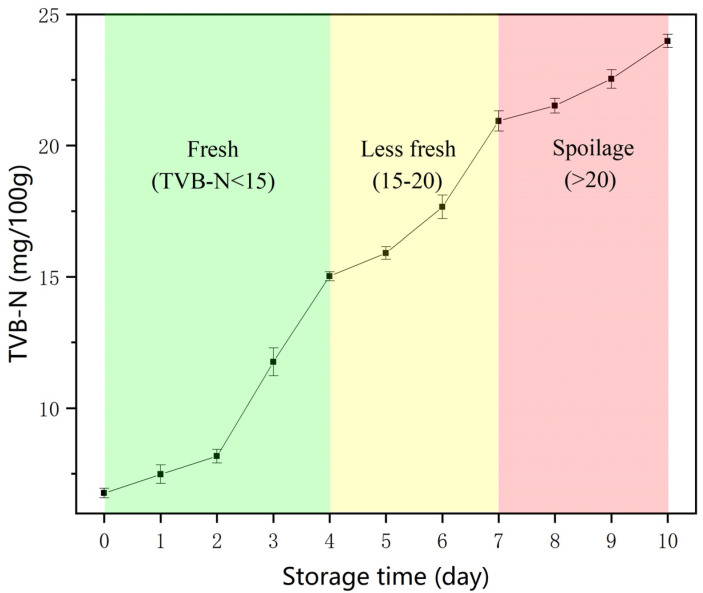
Changes in TVB-N for packaged chilled broiler meat stored at 4 °C.

The *R*, *G*, and *B* values of the colorimetric sensing label showed significant decreases when the colorimetric sensing label was used to monitor chilled broiler meat spoilage (the weight of each sample was 500 ± 2 g) (Figure 5e–g). Over time, the physicochemical parameters of packaged chilled broiler meat related to their quality, such as TVB-N content, underwent changes. The concentrations of TVB-N in the packaged headspace were monitored with respect to storage time. To classify the data from the colorimetric sensing label, the total volatile basic nitrogen (TVB-N) values of the chilled broiler meat were compared. For a 100 g sample, meat with a TVB-N value of ≤15 mg is considered fresh, 15–20 mg is edible but must be cooked, and ≥20 mg indicates spoiled meat [35,36]. As shown in Figure 5, every colorimetric Ag^+^ probe exhibited observable color changes. When stored under the same conditions, the initial TVB-N value of the meat (per 100 g) was 6.77 mg and exceeded 15 and 20 mg on days 4 and 7, respectively (Figure 6). As shown specifically for days 4 and 7 in Figure 5, there was a sufficient color change indicating the transition from fresh to edible (cooked) to spoiled meat. The colorimetric sensing label showed a suitable response to the changing quality of the chilled broiler meat. Furthermore, the visual inspection did not indicate any differences in the colors for blank samples (pure water) stored at 4 °C. Although several signal-based fluorescence, ratiometric fluorescence, voltammetry, or SERS methods (Table S1, Supporting Information) also present effective concentration ranges for the detection of amines, they have poorer visual effects than colorimetry methods because they are not easily distinguishable in the probe by the naked eye. Overall, this study demonstrated that the designed colorimetric sensing label is highly promising for use as a real-time freshness monitor. Consumers or retailers could assess the freshness of chilled broiler meat with the naked eye.

## 4. Conclusions

In summary, we reported a Ag NP-based colorimetric Ag^+^ probe as a new diverse and fast colorimetric label operating via chemical interactions with amines. The self-assembly reaction between colorimetric Ag^+^ probes prepared from sugar-stabilized Ag^+^ and amines produced by meat yielded Ag NPs and the easily biofunctionalized colorimetric Ag^+^ probe showed distinct color changes for different amines. The colorimetric Ag^+^ probe was combined with a colorimetric tank to produce a solid version of the colorimetric sensing label. The developed colorimetric sensing label responded to changes in the freshness of packaged chilled broiler meat and performed reliably in recording freshness history at different temperatures. The colorimetric sensing label produced irreversible color changes and operated on a timescale similar to that used in existing TVB-N monitoring. Since the D-ribose used are food additives (with excellent biocompatibility and meat safety), this novel colorimetric sensing label is nontoxic and eco-friendly. Hence, this colorimetric sensing label could be applied to a wide range of meat packaging systems and may lead to significant improvements in current meat packaging.

## Data Availability

The data presented in this study are available in Supplementary Materials.

## Supplementary Materials

The following supporting information can be downloaded at: https://www.mdpi.com/article/10.3390/nano12091389/s1, Figure S1. Absorbance at 440 nm for the reaction mixture after different incubation times (0, 0.5, 1, 2, 3, 4 and 5 h). Figure S2. Preparation of the colorimetric sensing label: (a) CAD top-side view of the colorimetric gel tank; (b) entity graph of the colorimetric gel tank; it was cut from foam tape with a thickness of 2 mm. There was one round holes with diameters of 10 mm in the middle, and the capacity was approximately 0.16 mL. The bottom was sealed with Tyvek paper, which has excellent breathability and safety. (c) Sample graph of a colorimetric sensing label. The colorimetric Ag^+^ probe was added to the round holes, and a 6 mm diameter black paper was pasted at the bottom right as the black background. Table S1. Comparison of the analytical performance of methods used for amine detection [41,42,43,44,45,46,47,48,49,50,51,52,53,54,55,56].
