# Supplementary material for: A Colorimetric Ag+ Probe for Food Real-Time Visual Monitoring
Source: Nanomaterials (Basel). 2022 Apr 19;12(9):1389. doi: 10.3390/nano12091389 (PMC9101572; doi:10.3390/nano12091389)
Supplement: Supplementary file 1 [file nanomaterials-12-01389-s001.zip › nanomaterials-1669999-supplementary.pdf]

Supplementary Materials

A Colorimetric Ag^+ Probe for Food Real-Time Visual Monitoring

Jiahang Yu, Jun Qi, Zhen Li, Huixin Tian and Xinglian Xu *

Jiangsu Collaborative Innovation Center of Meat Production and Processing, Quality and Safety Control,
College of Food Science and Technology, Nanjing Agricultural University, Nanjing 210095, China; 2019208009@njau.edu.cn (J.Y.); junqi86@hotmail.com (J.Q.); 2018808149@njau.edu.cn; (Z.L.); hxtian98@163.com (H.T.)

* Correspondence: xlxus@njau.edu.cn

Abstract: Monitoring food quality throughout

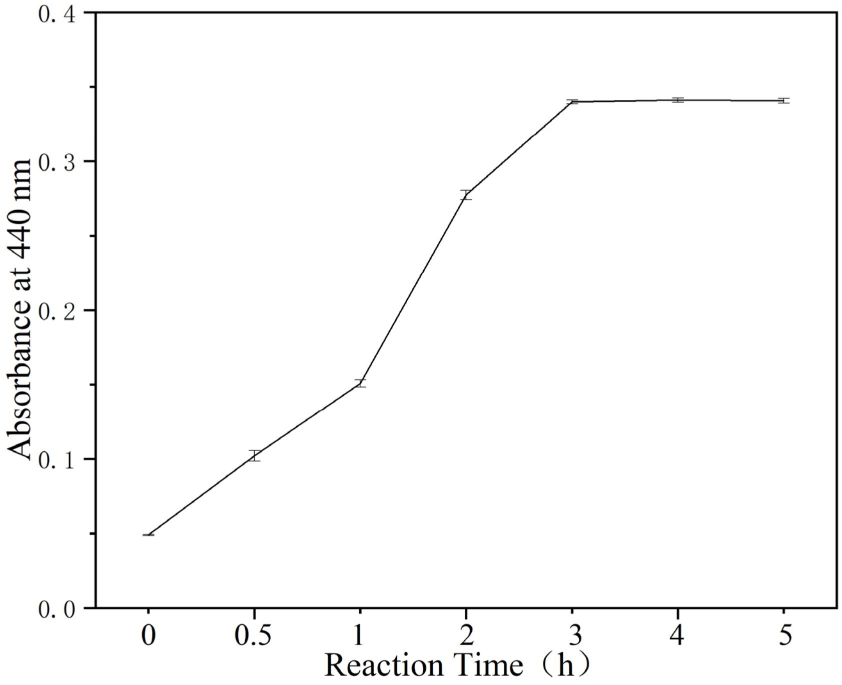

Figure S1. Absorbance at 440 nm for the reaction mixture after different incubation times (0, 0.5, 1, 2, 3, 4, and 5 h).

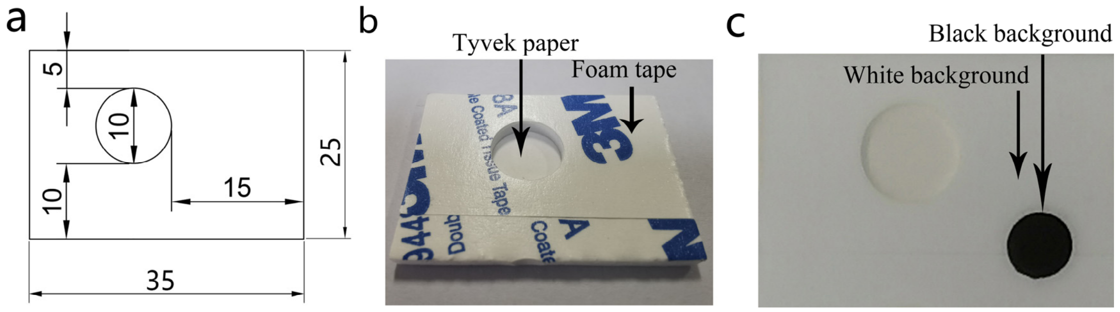

Figure S2. Preparation of the colorimetric sensing label: (a) CAD top-side view of the colorimetric gel tank; (b) entity graph of the colorimetric gel tank; it was cut from foam tape with a thickness of 2 mm. There was one round hole with a diameter of 10 mm in the middle, and the capacity was approximately 0.16 mL. The bottom was sealed with Tyvek paper, which has excellent breathability and

safety. (c) Sample graph of a colorimetric sensing label. The colorimetric Ag+ probe was added to the round holes, and a 6 mm diameter black paper was pasted at the bottom right as the black background.

Table S1. Comparison of the analytical performance of methods used for amine detection.

**Target analytes**	**Methods**	**Probes**	**Linear range**	**LOD**	Ref.
**NH3**	**Colorimetry**	**Ag+ probe**	**2-100 μM**<br>0.07-3.5 mg/L	**2 μM**<br>0.07 mg/L	This work
Putrescine	Colorimetry	Ag+ probe	2-100 μM<br>0.322-16.1 mg/L	2 μM<br>0.322 mg/L	
Cadaverin	Colorimetry	Ag+ probe	2-100 μM<br>0.35-17.51 mg/L	2 μM<br>0.35 mg/L	
Methylamine	Colorimetry	Ag+ probe	5-100 μM<br>0.338-6.75 mg/L	5 μM<br>0.338 mg/L	
Trimethylamine	Colorimetry	Ag+ probe	5-100 μM<br>0.296-5.91 mg/L	5 μM<br>0.296 mg/L	
Tyramine	Colorimetry	Ag+ probe	5-100 μM<br>0.686-13.72 mg/L	5 μM<br>0.686 mg/L	
NH3	Voltammetry	CSFET	0.01-0.1 mg/L	0.01 mg/L	[41]
	Voltammetry	PTS-PAni	5-40 ppm	5 ppm	[42]
	Voltammetry	Nanotube/Metalloporphyrin	0.5-20 ppm	0.5 ppm	[43]
	Ratiometric fluorescence	$Zn_2(bpdc)_2(bpee)$	0.28-10 mg/L	-	[44]
	Fluorescence	SNNU-88	5-100 mg/L	-	[45]
	Fluorescence	FJU-56	0-10 mg/L	1.38 mg/L	[46]
Dimethylamine	Voltammetry	MIP	0.0005-0.01 mol L^{-1}	0.0005 mol L^{-1}	[47]
Trimethylamine	Voltammetry	MnPc	0.1-0.6 ppm	0.1 ppm	[48]
Putrescine	Ratiometric Fluorescence	PFTBTCOOH/CS-graft-OA	0-10.12 mg/L	2.02 mg/L	[42]
	Fluorescence	OPA	0.07-17.63 mg/L	0.039 mg/L	[49]
Histamine	Fluorescence	CdSe/ZnS QDs	0.45–2.25 mM	0.11 mM	[50]
	SERS	AuNPs	0.027–0.80 mM	0.027 mM	[51]
	SERS	AgNPs	0–1.80 mM	-	[52]
	Voltammetry	Carbon nanotubes	4.5–720 μM	3.78 μM	[53]
	Voltammetry	AgNPs	1–500 μM	0.049 μM	[54]
	SERS	SiO_2@Au@Ag	0.1-0.8 mM	0.1 mM	[55]
	Ratiometric Fluorescence	EuMOF-FITC	2.78—41.68 mg/L	1.11 mg/L	[56]
